# Management of immune thrombocytopenia in multiple sclerosis patients treated with alemtuzumab: a Belgian consensus

**DOI:** 10.1007/s13760-018-0882-3

**Published:** 2018-01-27

**Authors:** Catherine Lambert, Benedicte Dubois, Dominique Dive, Andreas Lysandropoulos, Dominik Selleslag, Ludo Vanopdenbosch, Vincent Van Pesch, Bart Vanwijmeersch, Ann Janssens

**Affiliations:** 1 Department of Hematology, Cliniques Universitaires St Luc, Brussels, Belgium; 2 Department of Neurology, Universitaire Zienkenhuis Leuven, Leuven, Belgium; 3 Departement of Neurology, CHU Liège, Esneux, Belgium; 4 Neuroimmunology - MS Unit, Neurology Department, CUB- Hôpital Erasme, Brussels, Belgium; 5 Department of Hematology, AZ Sint-Jan, Brugge, Belgium; 6 Department of Neurology, AZ Sint Jan, Brugge, Belgium; 7 Department of Neurology, Cliniques Universitaires St Luc, Brussels, Belgium; 8 Departement of Neurology, Rehabilitation and MS Center, Overpelt, Belgium; 9 Department of Hematology, Universitaire Ziekenhuizen Leuven, Leuven, Belgium

**Keywords:** Alemtuzumab, Multiple sclerosis, Immune thrombocytopenia, Platelet count, Practical recommendations

## Abstract

Alemtuzumab (Lemtrada^®^) is a humanized monoclonal antibody indicated for the treatment of adult patients with relapsing–remitting multiple sclerosis with active disease defined by clinical or imaging features. Alemtuzumab demonstrated superior efficacy over active comparator in both treatment naive patients and those with inadequate response to prior therapy. Alemtuzumab is associated with a consistent and manageable safety and tolerability profile. Treatment with alemtuzumab for multiple sclerosis increases the risk for autoimmune adverse events including immune thrombocytopenia (ITP). Complete blood counts with differential should be obtained prior to initiation of treatment and at monthly intervals thereafter for 48 months after the last infusion. After this period of time, testing should be performed based on clinical findings suggestive of ITP. If ITP onset is confirmed, appropriate medical intervention should be promptly initiated, including immediate referral to a specialist. This paper presents the consensus of Belgian multiple sclerosis specialists and hematologists to guide the treating physician with practical recommendations.

## Introduction

Alemtuzumab (Lemtrada^®^) is a humanized monoclonal antibody approved in more than 60 countries. Within the European Union, alemtuzumab is indicated for the treatment of adult patients with relapsing–remitting multiple sclerosis (RRMS) with active disease defined by clinical or imaging features.  Lemtrada® is not recommended for patients with inactive disease or those stable on current therapy. Alemtuzumab demonstrated superior efficacy over active comparator in both treatment naive patients and those with inadequate response to prior therapy. Alemtuzumab is associated with a consistent and manageable safety and tolerability profile [1]. The most recent efficacy data over 6 years on clinical and MRI lesion activity as well as on brain volume loss suggest that alemtuzumab may provide a unique treatment approach for RRMS patients, offering durable efficacy in the absence of continuous treatment [2].

Treatment with alemtuzumab for multiple sclerosis (MS) increases the risk for autoimmune adverse events including immune thrombocytopenia (previously known as immune thrombocytopenic purpura) [3–5].

A first case of ITP after alemtuzumab occurred unexpectedly in the phase 2 study in MS and resulted in a fatal outcome [4]. A risk management plan (RMP) put in place ensured early detection of symptoms or signs of autoimmune disease, with the aim of minimizing the impact of alemtuzumab-associated autoimmune effects.

The European risk management plan includes complete blood counts with differential which should be obtained prior to initiation of treatment and at monthly intervals thereafter for 48 months after the last infusion. After this period of time, testing should be performed based on clinical findings suggestive of ITP. If ITP is suspected, a complete blood count should be obtained immediately. At the time of treatment with Alemtuzumab, the patient should be educated to remain vigilant for bleeding symptoms [6–8]. In the event of an abnormal platelet count the sequence of additional tests and the appropriate moment to refer the patient to a hematologist will be at the discretion of the treating physician. If ITP onset is confirmed, appropriate medical intervention should be promptly initiated, including immediate referral to a specialist. This paper presents the consensus of Belgian MS specialists and hematologists to guide the treating physician with practical recommendations.

## Alemtuzumab and ITP

ITP after receiving alemtuzumab has been described as a specific form characterized by delayed onset, responsiveness to conventional ITP therapies, and prolonged remission [5].

**Fig. 1:**
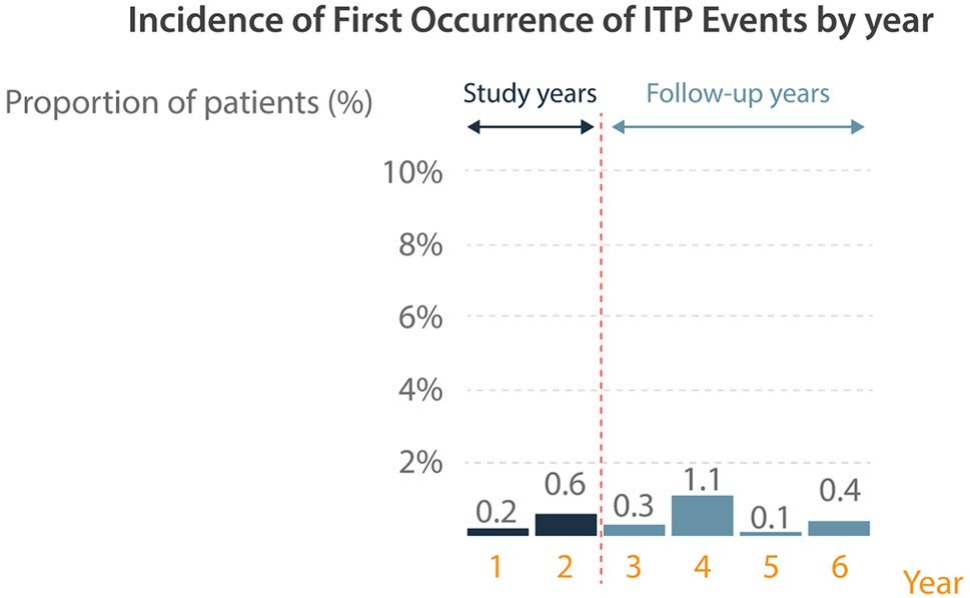
Incidence of first occurence of ITP by year in the CARE-MS studies

Autoimmune adverse events were detected in MS patients treated with alemtuzumab in clinical trials [8]. The 6-year follow-up data of the CARE-MS studies were presented at ECTRIMS 2016 and showed the following frequencies: 39% of alemtuzumab treated subjects experienced an autoimmune thyroid disorder, 2.6% an immune thrombocytopenia and 0.2% (two cases) an autoimmune renal disease. The incidence of first occurrence of ITP by year is shown in Fig. 1 [9].

From all cases of thrombocytopenia detected in the phase 3 trials, 80% was by monthly blood monitoring and 20% by patients’ recognition of clinical symptoms [10].

In post-marketing use through February 2017, 13,000 patients have been treated worldwide with alemtuzumab for MS and the frequency for ITP has been estimated at 0.58% [11]. Post-marketing frequencies are not directly comparable to clinical trial incidences because of differences in ascertainment methodology and follow-up duration, and limitations of post-marketing reporting.

## Recommendations for the follow up of platelet counts in patients treated with alemtuzumab

### Before starting treatment with alemtuzumab

Complete blood count (CBC) with differential should be obtained prior to initiation of Alemtuzumab (treatment and pre-phase with steroids) [7].

There are no data available about initiation of alemtuzumab in patients with low platelet count.

### Once treated with alemtuzumab: monitoring of platelet count

Complete blood count with differential should be obtained at monthly intervals thereafter for 48 months after the last infusion. After this period of time, testing should be performed based on clinical findings suggestive of ITP. If ITP is suspected a CBC should be obtained immediately [7].

### Bleeding risk and platelet count

**Fig. 2:**
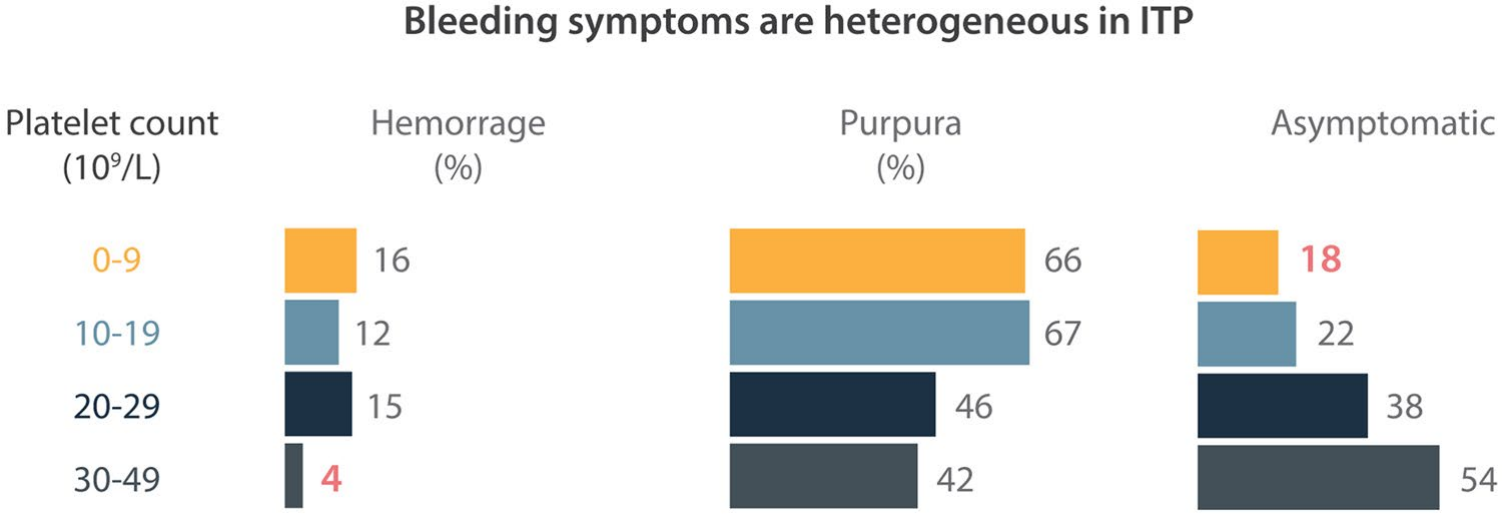
Heterogeneity of bleeding symptoms in ITP

It’s also important to realize that there is no linear relationship between platelet count and bleeding symptoms. However, severe bleeding usually occurs with a platelet count below 10,000/μl Fig. 2 [12, 13].

If ITP onset is confirmed, appropriate medical intervention should be promptly initiated, including immediate referral to a specialist. The following consensus goes beyond alemtuzumab’s SmPC and aims to guide treating physicians on the necessary actions in case of an abnormal platelet count and before ITP is confirmed. Reference is made to the guidelines elaborated by the Belgian Hematological Society (BHS) for treating primary ITP in adults [14].

#### Education of the patients

It is imperative to educate the patient to be vigilant for any clinical sign suggestive of bleeding between the monthly CBC checks. In case of such a sign, the CBC must be obtained immediately.

**Fig. 3:**
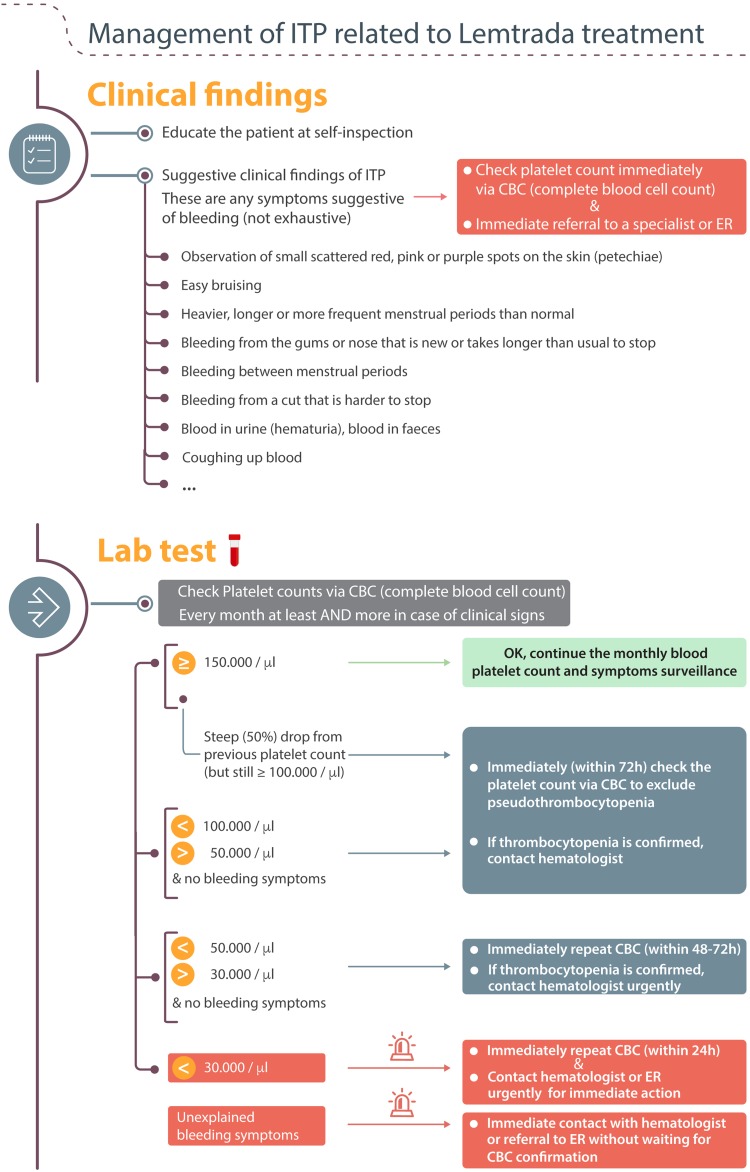
Values for referral and diagnostic work-up

These suggestive clinical findings are described as follows: the observation of small scattered red, pink, or purple spots on the skin (petechiae); easy bruising; bleeding from a cut that is harder to stop; heavier, longer or more frequent menstrual periods than normal; bleeding between menstrual periods; bleeding from the gums or nose that is new or takes longer than usual to stop; or coughing up blood. Any of these should prompt further action including immediate referral to a specialist [7] (Fig. 3).

#### Values for referral and diagnostic work-up

Figure 3 describes which action to undertake depending on the platelet count received after every CBC check.

- When the platelet count is at least 150,000/μl, continue the monthly blood platelet count and bleeding symptoms surveillance.
- Any steep decrease of 50% or more from previous value but still above 100,000/μl must prompt an immediate recheck of the CBC to exclude pseudothrombocytopenia (platelet aggregates). Contact the hematologist when this steep decrease is confirmed.
  - Pseudothrombocytopenia (platelet aggregates) has to be excluded by checking the platelets on EDTA, heparin, or citrate anticoagulated blood. A peripheral blood smear must exclude platelet clumping or aggregation.
- When the count is below 100,000/μl and still above 50,000/μl and with no bleeding symptoms, check the platelet count via a new CBC within 72 h to exclude pseudothrombocytopenia. If thrombocytopenia is confirmed, contact the hematologist to discuss referral.
- With a value between 30,0000 and 50,000/μl and no bleeding symptoms, recheck the CBC within 48–72 h to exclude pseudothrombocytopenia and contact the hematologist after the results have been confirmed.
- Below 30,000/μl, recheck the CBC and contact the hematologist immediately.
- When bleeding symptoms are present, contact the hematologist immediately for urgent referral or send the patient to the emergency unit.

#### Further diagnostic work up and values for treatment of ITP

**Fig. 4:**
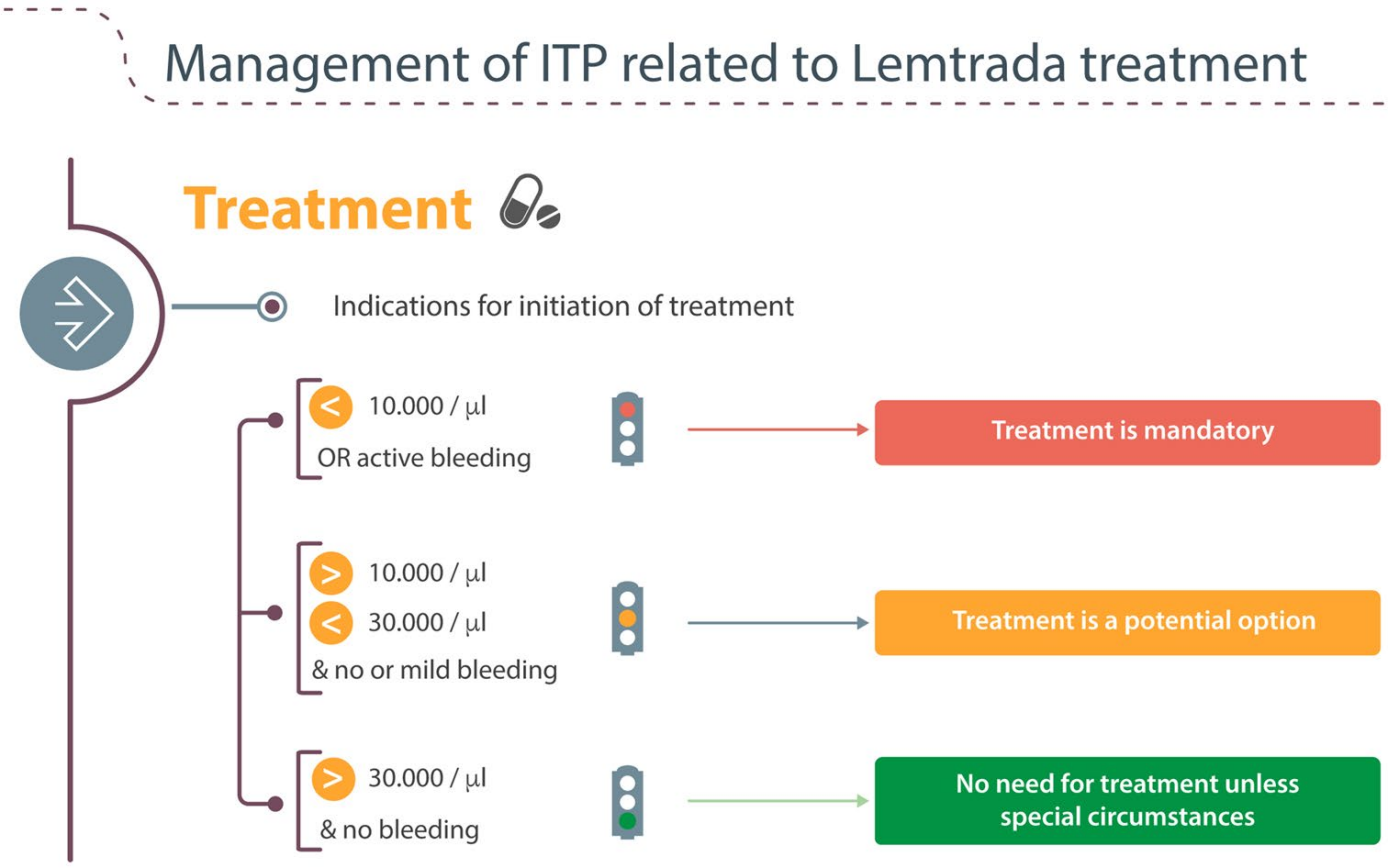
Platelet values for the initiation of treatment in ITP

The further diagnostic work up for a suspected ITP has been well described in the publication of the practice guidelines by the BHS [14]. These guidelines also set clear values for the initiation of treatment (Fig. 4) and the therapeutic options.

Active bleeding or platelet count below 10.000/μl make treatment mandatory. When there’s no or mild bleeding and the platelet count is between 10 and 30,000/μl, then treatment is a potential option after evaluation of patient characteristics.

When there is no bleeding and platelet count is above 30,000/μl then there is no need for treatment unless special circumstances are present (e.g., invasive procedure).

The decision to hospitalize a patient with ITP will be made by the hematologist primarily based on the hemorrhage situation (patients with signs of mucosal or deep bleeding are frequently admitted) and on factors influencing the bleeding risk (age, comorbidities, need for treatment with anticoagulant or antiplatelet agents, etc.) [14].

## Conclusion

The use of alemtuzumab has been associated with the development of ITP in 2, 6% of patients in clinical trials in MS. A lower frequency was reported in the post-marketing setting. Through the RMP, patients can be diagnosed early, and treated if needed, allowing for favorable outcomes. This important goal can be reached through a good education of the patient, a careful watch on the monthly lab tests and a close collaboration between the neurologist and the hematologist. The latter could be facilitated through the establishment of a reference network prior to initiation of alemtuzumab treatment. In this report, based on a consensus meeting with Belgian hematologists and neurologists, we have described different scenarios of platelet count abnormalities that can be encountered and the appropriate actions to take for every scenario.
